# Abstracts and Gleanings

**Published:** 1882-06-20

**Authors:** 


### ABSTRACTS AND GLEANINGS.

**The Parasitic Nature of Tubercular Consumption.**—Professor Tyndall has communicated to the London Times an account of results obtained by Dr. Koch, of Berlin, the investigation of the etiology of tubercular disease, as set forth by him in an address delivered March 24th, before the Physiological Society of Berlin.

It was the aim of Dr. Koch to determine the precise character of contagion which previous experiments on inoculation and inhalation had proved to be capable of transferring, and reproducing tubercular consumption.

In pursuing these investigations, Dr. Koch subjected the diseased organs of a great number of men and animals to microscopic examination, and found, in all cases, the tubercles infested with a minute rod-shaped parasite, which, by means of a special dye, he differentiated from the surrounding tissue. It was, he says, in the highest degree impressive to observe in the centre of the tubercle cell the minute organism which had created it. Transferring directly by inoculation the tuberculous matter from diseased animals to healthy ones, he in every instance reproduced the disease.

To meet the objection that it was not the parasite itself, but some virus in which it was imbedded in the diseased organs, that was the real contagium, he cultivated his bacilli artificially for long periods of time, and through many successive generations. With a speck of matter, for example, from a tuberculous human lung he infected a substance prepared, after much trial, by himself, with the view of affording nutriment to the parasite. Here he permitted it to grow and multiply. From this new generation he took a minute sample, and infected therewith fresh nutritive matter, thus producing another brood. Generation after generation of bacilli were developed in this way without the intervention of disease. At the end of the process, which sometimes embraced successive cultivations, extending over half a year, the purified bacilli were introduced into the circulation of healthy animals of various kinds. In every case inoculation was followed by the reproduction and spread of the parasite, and the generation of the original disease.

In the course of his experiments Dr. Koch determined the limits of temperature between which the tubercle bacillus can develop and multiply to be 60° Fah., and a maximum of 104°.

He concludes that, unlike the bacillus anthracis of splenic fever, which can flourish freely outside the animal body in the temperate zone, animal warmth is necessary for the propagation of the newly discovered organism.

In a vast number of cases, Dr. Koch has examined the matter expectorated from the lungs of persons affected with phthisis, and found in it swarms of bacilli, while in matter expectorated from the lungs of persons not thus affected he has never found the organism. The expectorated matter in the former cases was highly infective, nor did dying destroy its virulence. Guinea pigs infected with expectorated matter which had been kept dry for two, four and eight weeks respectively, were smitten with tubercular disease quite as virulent as that produced by fresh expectoration. Dr. Koch points to the grave danger of inhaling air in which particles of the dried sputa of consumptive patients mingles with dust of other kinds.

Commenting upon this important communication from Prof. Tyndall, the London Times points out the significant fact that though the experiments of Dr. Koch seem as yet to have been carried no further than to the repeated cultivation of the tubercle bacillus in its original virulence, they will speedily be followed, as a matter of course, by attempts at cultivation in diminished intensity. The evidence even now, the Times continues, does not rest upon the labors of Dr. Koch alone, for Prof. Klebs, five years ago, declared the infective property of tubercle to be due to the presence of a microphyte (practically a synomym for bacillus), and Dr. Schuller, of Griefswald, a resume of whose investigations was given by Mr. Simon to the International Medical Congress, has proved that the microphyte which characterizes tubercle characterizes also certain affections popularly called scrofulous, such as diseased joints and glands, and that inoculation from any of them, or with a fluid in which the microphyte has been cultivated, will infect with general tuberculosis. Dr. Schuller, according to the same authority, has also made proposals for the treatment of tubercle on the basis of its micro-parasitic origin, and has shown the successful results of such treatment upon animals which he has inoculated.—*Scientific American, May 13th.*

**Prolonged Gestation.**—In the May number of the New York Medical Journal and Obstetrical Review, Dr. Louis A. Rodenstein, of New York, reports four cases of prolonged gestation, and remarks that the number of cases cited upon undouted authority by every writer on obstetrics, and the cases constantly reported as occurring under the personal observation of general practitioners, go to show that prolonged gestation is not a myth, and especially that it should not be explained away by questioning the virtue of the mother. How long the duration of the period of gestation can extend beyond the normal time is not yet determined, perhaps cannot be determined, but that it may extend over two months is apparently settled. The same principle is involved, whether the uterus tolerates the presence of the child three days or one hundred and forty-five days (Prof. Meigs' Report) after the natural term of gestation has expired. He believes that, after the uterus has performed its physiological function of gestation for the natural term, it rests from the work of gestation proper. Why does it not, then, exercise the function of expulsion? That question he does not attempt to answer, but believes that after gestation has performed its proper and peculiar work the growth of the child is complete, and it thereafter lies dormant in the womb. Otherwise the child would grow to huge size, and its delivery in the natural way would be impossible; whereas in the case cited the size of the child at the expiration of the period of prolonged gestation was normal.—*Detroit Clinic.*

**Vehicle for Salicylic Acid.**—I have been, for three years past, using the following: ℞.—Salicylic acid, ½ ℥; liq. acet. ammonia, ℥ij; syr. limonis, ℥ij; aquæ, ℥ij. M. Sig.—One teaspoonful every hour.

The amount of salicylic acid can be doubled in case it is necessary, without changing the amount of the other ingredients, and given as frequently. Sometimes the syrup of lemon is not at hand, and then I proceed thus: ℞.—Salicylic acid, ℥i; liq. acet. ammonia, ℥viij; aquæ, ℥viij. M. Sig.—One teaspoonful every two or three hours.

As effervescence takes place when adding the acet. ammonia, it is best to make the mixture in some broad, tolerably deep vessel, and after subsidence pour it off. This is a most eligible preparation, as the taste is effectually concealed, and it is, in fact, quite pleasant to take. By having the ammonia in the mixture, we subserve an important end, viz: the prevention of the formation of heart clots, which, it is said, is not of unfrequent occurrence in rheumatism, and the detachment of which produces such dire and often fatal effects. Instead of the traditional "six weeks in bed," I usually have my patients able to hobble about in from four to eight days. One case in particular, in which all the joints of the lower extremity, and all of the upper, except the left shoulder, elbow and wrist, were intensely swollen, so that it was inpossible for him to move in bed, yielded so rapidly to the effects of the remedy, that at the end of four days he could hobble about on crutches, and I discharged him on that day. As this was a severe case, I gave a tablespoonful of the second prescription every two hours. These prescriptions are not original with me, and, if I remember aright, I copied them from the Brief some years ago, but the brethren will find them as reliable as I have.—*Wm. W. Moore, M.D., in Medical Brief.*

**Treatment of Hay-Fever.**—Dr. Herman Hager, who has observed a case of catarrh with subsequent asthmatic trouble and loss of appetite, which closely resembled the hay-fever of England and the United States, thought of trying his catarrh-pills (1), prepared after the following manner:

| | | | |
|---|---|---|---|
| ℞ | Quinidiæ sulphatis | 10.0 | gm. |
| | Tragacanthæ | 4.0 | " |
| | Althæa rad. | 1.0 | " |
| | Gentian. rad | 8.0 | " |
| | Glycerin | 7.0 | " |
| | Acid. hydrochloric. | 7.0 | " |

M. Make 200 pills. Take three every two hours.

(Comp. also New Remedies, 1880, 243; 1881, 254.)

The condition of the patient improved in the course of the first day, and on the second day the patient was well. Six months afterwards the attack again occurred, but yielded readily to the same treatment.

Dr. Hager thinks that hay-fever is caused more probably by the dust or spores of fungi, than by the pollen of phænogamous plants.—*Pharm. Centralhalle, No. 18.*

**Effect of an Overdose of Podophyllin — amount taken about sixty centigrams (ten grains).**—Prof. D. W. Prentiss reports the following case in the Medical Times: Mrs. H., aged about 45 years, a strong, healthy person, had been constipated for a week, and was feeling badly in consequence. Her husband was in the habit of taking podophyllin for constipation, and had a bottle of it in the house. Mrs. H., knowing this circumstance, got the bottle, and took out as much of the medicine as could be held on the handle of a teaspoon, mixed it with a little water, and swallowed it. The dose was taken April 9, at 5 p.m.

At 7 p.m. had cutting pains on both sides of the abdomen, with desire for stool.

At 8 p.m. feeling very badly, went to bed. The pain had ceased; there was great exhaustion, with relaxed muscles and a feeling as though the body was bathed in sweat, which it was not; then came a fearful pain in the occiput, as "though the head was being split open." This pain lasted about two minutes, and was followed by a dull throbbing ache and feeling of heaviness, so that the head could not be raised from the pillow. At 8:30 o'clock vomiting began—first the contents of the stomach, then thin, bitter, dark-green fluid—from half a pint to a pint at each attack. There were six or seven spells of vomiting between 8:30 o'clock and 4 o'clock the next morning. With eaeh spell of vomiting the bowels moved—first constipated, then thin, watery stools, but no blood. There was no pain with the stools. Frequent sensations of heat passing over face and head were noticed. With each occasion of vomiting the exhaustion was so great that she felt as though dying. Could not raise the head or assist in the act of emesis.

I was called to the case at one o'clock in the night—eight hours after the podophyllin had been taken—when I found the patient in a state bordering on collapse: features pinched, extremities cold, pulse very feeble. Administered hypodermic injection of morphia sul. 1 centigram (gr. 1/6), atropia sul. ½ milligram (gr. 1-120) and followed it by sherry wine and lime-water, equal parts, small tablespoonful every fifteen minutes. Also left some morphia and atropia powders as above, to be taken every two hours if required. Hot applications to abdomen and extremities. There were two attacks of vomiting in the night after my visit, but much less severe, and on the morning of the 10th of April the patient was all right again, except the exhaustion.

**Therapeutic Effects of Damiana.**—In Paris Medical, No. 48, we read that damiana, turnera aphrodisiaca, is a herbaceous plant belonging to the portlaca family, and growing in Brazil and on the western coast of Mexico. Its flowers are white, and have the odor of buchu. It is gathered during August, while its stems are covered with an odoriferous gum-resin.

Damiana has for a long time been used among the Mexicans as a tonic; its stems and leaves are made into a decoction, which is taken to restore strength and nerve energy. It is also given in cases of impotency in either sex. It exerts a specially tonic and stimulating effect on the genito-urinary organs of both sexes, and when given in medium doses, acts as an aphrodisiac, an alterative, and a laxative, with a tendency to increasing the urinary secretions and developing sexual desires. In small doses, it appears to have specific tonic effects on all the pelvic organs, and gives increased activity to the secretions. It is also recommended as a nervine.

Damiana could, therefore, be used as an aphrodisiac in spermatorrhœa, in atrophy of the testicles, and in incontinence of urine; also as a powerful stimulant of the cerebral faculties, and in all accidents attending premature labor, in difficult menstruation, and in the diseases following gestation.

The effects of damiana are quite different from those of strychnine, phosphorus, or cantharides, which are given in small doses, for the purpose of obtaining immediate results. It acts, not as an irritant, but as a stimulant of the brain, and a tonic of the nerve centres governing the urino-genital apparatus, and its use has to be continued for several weeks. Its effects are chiefly noticeable on the sympathetic nerve, and when taken in large doses, it produces a peculiar intoxication, attended with slight pains in the prostatic region. Its good effects on the kidneys, the bladder, and the genital organs, are also manifest.

In doses of a teaspoonful three or four times daily, continued for several days, damiana has a pleasant laxative effect. The fluid extract is generally prescribed; it is combined with equal parts of pure glycerine, or syrup of tolu, or a fruit syrup of some kind. It may also be taken in wine. It is given in doses of thirty to sixty grains, three or four times daily. A solid extract is also prepared, of which the dose is thirty to sixty centigrams, (=gr. iv ss—gr. ix). *Med and Surg. Rep.*, Feb. 18, 1882.

**Vomiting in Pregnancy.**—Dr. Crounse in Medical Annals, reports a case of obstinate vomiting in pregnancy which, having resisted many remedies, was relieved as follows—he remarks : She now insisted so strongly upon having an operation for abortion performed that I concluded to pretend to accede to her wishes, and accordingly introduced the speculum, and with a uterine sound (pointed) slightly irritated the os, intending, under cover of this, to try still further the effect of other remedies. I left her entirely satisfied, thinking the desired operation had been performed. Judge of my surprise upon being told at my next visit that the vomiting had been very slight, that she had taken and retained some liquid food, and the drooling had, in great measure, subsided. She continued in this condition four days, when the vomiting returned. I again introduced the speculum and irritated the os, this time so that a slight trace of blood was visible. The vomiting again subsided, and she was able to take and retain food both liquid and solid. There was no return of the vomiting for two weeks, when it began again, but subsided upon irritating the os as before, and thus it continued, with occasional attacks, each of which readily disappeared by the same irritating process. After the sixth month there was no return of the vomiting, and at the end of gestation I delivered her of a female child weighing 8¾ pounds.

**Bandage After Delivery.**—In Wisconsin State Medical Society Dr. F. H. Day, of Wanwatosa, read a paper advocating and defending the use of the binder after confinement, as a support to the recently overdistended abdominal walls, controlling excessive mobility of the uterus, and aiding in the prevention of hemorrhage. The Doctor declared that he would no sooner neglect the universal application of the bandage than neglect bandages and splints for a fractured limb. He would have the bandage worn in many cases for weeks, or even months, and would hold any practitioner to be criminally negligent who was careless or indifferent as to when or how it was applied. He believed the bandage useful also in preserving the form of the woman.

Dr. Hunt said that, though he had been taught that the bandage was an invaluable aid in preventing after pains, and in diminishing the danger from flooding, and though he had used it with those ideas during the first ten years of his practice, he had applied it but rarely for the last twelve or thirteen years, except in cases where the patient had ventral hernia, or was very corpulent. He did not believe it could be applied tightly enough to have any effect on bemorrhage without causing great discomfort. He always applied firm pressure over uterus during removal of placenta, and for five or ten minutes afterwards, which in most cases, insured permanent contraction.

Dr. Griffin spoke particularly of the theory that the binder was useful in restoring and preserving the symmetry of the form, decidedly dissenting from that opinion. He believed the binder useful as a temporary support to the recently distended and now tired abdominal walls, but that its use was limited to a very short time, and that in most cases it became a discomfort and a nuisance to a woman after a very few days.

Dr. Vivian believed the bandage wholly useless, except for the temporary feeling of comfort it gave the mother.

**Typhoid Diarrhœa.**—Upon admission patient was somewhat prostrated; has diarrhœa and tenderness in the epigastric region; the tongue is slightly coated in centre, moist; appetite poor; pulse slow; no fever. Heart, lungs and urine normal. Ordered quinine gr. viij. daily. Hope's camph. mixt. f. ℥ss. as needed, and liquid diet.

April 20th. Diarrhœa has been checked, and he feels much better.

April 25th. Feels very well; bowels regular; appetite good. Temperature has ranged from 98° F. to 98.5° F.

CASE V. Diarrhœa with typhoid prostration. M. Z., aged 29, born in Germany, laborer; admitted April 17, 1882; discharged April 25, 1882, cured. Eight days ago, while working at a sugar refinery, he began having diarrhœa and pain in the bowels, and these have continued up to the present time.

Upon admission his face is slightly flushed, and he has a typhoid appearance; is somewhat prostrated; tongue coated in centre, moist; pulse slow, bowels were opened six times during the night. He has no fever, and there is no fullness of the abdomen. Examination of heart, lungs and urine gives negative results. Ordered Hope's camphor mixture f ℥ss. to be given as needed, quinine gr. viij. daily, and liquid diet.

April 23d. Feels very well; bowels regular; no pain; appetite good. Temperature has ranged from 98° F. to 98.5° F.

CASE VI. Diarrhœa with typhoid prostration. M. W., aged 19, born in Germany, single, laborer; admitted April 24, 1882; discharged April 29, 1882, cured. Has generally been very healthy. Came to this country eight months ago; two weeks ago he began working at a sugar refinery; about two weeks ago he was taken with diarrhœa, which has since become worse; has not had any nausea or pain.

Upon admission patient is slightly prostrated; face flushed; temperature also subnormal, 98° F.; tongue slightly furred, moist; pulse slow; appetite poor; bowels loose. Examination of heart, lungs and urine gives negative results. Ordered Hope's camphor mixt. f ℥ss. to be given as needed.

April 29th. Diarrhœa has been checked, appetite has improved, and he seems very well again.—Dr. Hutchinson in *Boston Med. Journal.*

**Treatment of Acute Dysentery with Aconite**—Dr. William Owen (Indian Medical Gazette) reports one hundred and fifty-one cases of acute dysentery occuring in the Convict Hospital, Port Blair, India, which were treated with tincture of aconite. All the cases were typical examples of acute dysentery; and all, with one exception, recovered. He states that he was led to give aconite a trial, as the remedy most likely to be successful, from the following considerations: 1. From its beneficial action in other acute inflammations; 2. From its effects on the capillaries of the skin which it dilates, thus relieving internal congestion; 3. From its antipyretic action in febrile cases; 4. From its sedative action on the mucous membrane of the stomach and intestines, and its beneficial action in some forms of dyspepsia. In the first case in which he tried this remedy he was somewhat diffident, and he had ten cases in which a combined treatment of ipecac and aconite was used. However he soon discontinued the ipecac entirely, finding there was no occasion for its use.

Dr. Owen gives one minim every quarter of an hour for the first two hours, and a minim every subsequent hour, or thirty minims in twenty-four hours. This method he finds to be followed by the best results, inasmuch as the action of the medicine is more rapidly established, and an effect on the disease was more quickly produced than by the other methods.—*Med. News.*

**Liebig's Corn Cure.**—Extract cannabis indica 5 parts; salicylic acid 30 parts; collodion 240 parts; mix and dissolve. It is applied with a camel-hair pencil, so as to form a thick coating, for four consecutive nights and mornings. The Indian hemp acts as an anodyne, and the acid disintegrates the corn, so that after a hot bath on the fifth day it will come out, adhering to the artificial skin of collodion on the toe. This causes no pain and is said to be very effective.—*Mich. Med. News.*

**Rules of Practice in Operating upon Pregnant Women.**—Dr. A. Verneuil (International Surgery, vol I., p. 334) gives the following: Operate at once upon those affections which immediately endanger the life of the mother, and against which medical treatment would be certainly or almost unavailing.

Operate also at a suitable time, and after having tried palliative or curative remedies, in those diseases which, though not immediately compromising life, endanger it by their progress, and tend to become incurable if not met with energetic treatment. Operate also in those affections which without disturbing pregnancy and without being aggravated by it, become at its termination causes of dystocia. In these cases the surgeon may operate before or at the very period of delivery, upon the mother or upon the fœtus, the premature expulsion of which may be induced. An attempt should be made to save both mother and fœtus, but this being impossible the latter must be unhesitatingly sacrificed to the former.

Abstain as far as possible from every operation in those affections which are uninfluenced by pregnancy—and which in turn only compromise pregnancy and parturition indirectly—by as far as possible allowing nature to act, and by aiding her by mild measures.

Abstain absolutely from every operation for affections which compromise only the form or function of the organs of secondary importance, or which are susceptible of spontaneous cure after delivery.

Avoid, as far as possible, every operation during the puerperal state. In case of danger, operate rather during pregnancy, and under opposite circumstances, postpone interference until a period sufficiently remote from delivery.—*American Med. Weekly.*

**Cholera Infantum.**—Dr. S. Perry says, in American Medical Weekly, of cholera infantum : I used to be greatly troubled with this disease in my practice, and have tried most of the remedies suggested by medical writers; but with poor success. The remedy I find to answer the purpose, and worth all others combined, is sulphuric acid. After a dose of calomel and castor oil, the sulphuric acid, with a small quantity of quinine. In addition use starch and laudanum enemas. Whoever tries this remedy will not be ashamed of his suceess in the treatment of cholera infantum. I have tried sub-nitrate of bismuth, and many other articles. They are nothing compared with the acid treatment.

I have practiced medicine for thirty years and have seen many remedies, and almost as many failures.

**Antidote for Strychnine.**—The British Medical Journal of March 11, 1882, stated that Messrs. Greville, Williams & Waters of the Royal Society, have discovered an antidote for strychnine. The substance is named *lutidine*, and is obtained by distilling cinchonine with caustic potash. The efficacy of the remedy has been tested by experiments on frogs. The results of the experiments are most promising and lend encouragement to the hope that, at least, a reliable antidote has been discovered.—*Canada Lancet.*

**Treatment of Rabies with Hoang-Nan.**—Hoang-nan is a creeper found in the mountains sedarating the kingdom of Annam from Laos. This plant belongs to the family of Loganiaceæ. So says Dr. Lesserteur. It produces the same physiological effects as strychnia and brucia. However, under its influence, it is always the lower limbs which first are affected with tetanus, and in which the greatest amount of contraction is observed after death.

The Tonqua remedy is prepared after the following formula:—Alum, 1 part; native realgar, 2 parts; bark of hoang-nan, 2 parts; the whole is powdered and made into pills containing each 25 centigrammes of the mixture.

Hoang-nan alone and the above mixture have been used as remedies for the leprosy, rabies, bad ulcers, scrofulidæ, paralysis, etc. It is reported to have marvelous effects in leprosy, yet experimenters have not met with any success. It has the reputation of being infallible for rabies, even when in its worst period, but such affirmations have not scientific value.

The Tonqua remedy was known at the beginning of the last century; it was then composed of cinnabar and musk. It seems to have rendered good service in the hands of the physicians of the time, in the treatment of rabies.—*Paris Medical.*

**The Proper Dose of Conium.**—Seguin (Archiv. of Medicine, April, 1882), commenting upon the dose of this agent (he employs the fluid extract, Squibb), says that to get any effect from it we must use much larger doses than are usually recommended. He says use it in chorea, spasm or paralyzed limbs, general irritability, and insomnia. To obtain muscular relaxation as in chorea, after a few tentative doses of 20 and 40 minims, he gives 60, 80 or even 100 minims, which cause ptosis (sometimes diplopia) and paresis of arms and legs. He does not repeat until the effects have passed off—12 to 24 hours. He has almost perfectly cured a chronic adult chorea of 14 years duration by teaspoonful doses daily for a month or more. Many cases of insomnia with wakefulness in the first part of the night, more especially those with fidgets or physical restlessness are very much benefited by conium—m. xx with gr. xx bromide of potassium, to be repeated if necessary. The indications of conium can only be fulfilled by obtaining its physiological effects between which and the toxic effects there is a wide distance.—*Md. Med. Jour.*

**Relief of Pain in Lead Colic**—Dr. Geneuil, in a note to the Bulletin de Therapeutique, after alluding to the various means adopted for the relief of the terrible pains of lead colic, as rubefaction by synapisms, chloroform, electricity, and hypodermic injections of morphia, relates a case to which he was called in the country, where none of these means were at hand, and in which he succeeded in giving complete and permanent relief by a very simple procedure. Having directed a napkin to be heated at the fire, he first applied a towel wetted with almost ice-cold water to the whole surface of the abdomen, while the patient was shrieking with pain, and having retained it there for four or five seconds.

rapidly replaced, it by the almost burning napkin. The effect was like enchantment, the pain instantly disappearing and sleep following, without any return of suffering. The cause of the colic was at first obscure, but was found to depend upon the patient, who was an inveterate smoker, and had very often in the day to relight his pipe, which he did by means of matches colored with chromate of lead.—*N. Y. Med. Record.*

**Can a Man Have Syphilis Twice?**—The man whom we have just seen offers a remarkable example of the occurrence of a second chancre soon after the first. His second sore has been, as I have repeatedly demonstrated, characteristically indurated. He is quite candid, and made no doubt that this sore was the result of contagion; yet it is barely a year since he had his first chancre, and this was followed by an eruption, of which he had scarcely got clear when this second sore occurred. The case is proof that man may have an indurated sore on the penis within a year of the former one; but it is not proof that he may have syphilis twice; for this patient has not as yet had any constitutional symptoms as the result of the last chancre. If, however, you ask me for an answer to the general question, Can a man have true, complete syphilis twice? then I must reply clearly that he can. Such cases are rare—as rare, perhaps, as second attacks of small-pox—but they do occur.—*Cincinnati Lancet and Clinic.*

Unquestionably, a man may have syphilis again and again, and this fact is very strong as an argument in favor of the *curability* of the disease. Prof. Roswell Park, of Chicago, reports, in a recent number of the Southern Clinic, the case of a patient under his care with a second attack of syphilis.—[*Ed. Southern Clinic.*

**Wight on the Combating of Small-Pox.**—This is a paper read before the Ann Arbor Sanitary Convention. As its author is the present efficient and able health officer of Detroit as he formerly was of Milwaukee, we have read his paper with more than usual interest.

He shows that inoculation failed to be a satisfactory method of combatting small-pox.

He calls attention to several groupings of facts showing that vaccination furnishes the best method for fighting small-pox:

1. The great mass of enlightened people believe in it.
2. Educated medical men are almost unanimous in favor of vaccination.
3. The governments of nearly all civilized nations favor vaccination.
4. Statistics on a large scale demonstrate the utility of vaccination.
5. Special statistics also demonstrate the benefits of vaccination.

As to the degree of protection of vaccination when it is properly performed, it is as complete a proteetion as small-pox itself.—*Detroit Lancet.*

**Micro-Organisms and Disease.**—Dr. Declat, with the object of going straight at the source of the mischief, suggested the vigorous use of phenic acid, by injection into the veins and otherwise, as a means likely to prove effective against the organisms of yellow fever and kindred acute forms of malarial poisoning. The suggestion went out to Brazil, and in June last M. DeLacaille, a French physician resident in Rio, wrote home his experience of it. The first case in which he tried it was a young lady apparently on the point of death from the worst form of the disease—a fever attended with the fatal black vomit. In three days she was out of danger. "During the thirty years in which I have been employed in fighting yellow fever," writes M. De Lacaille, "this is the first patient whom I am certain of having snatched from death at such a period of the disease." In a dozen other cases the treatment was crowned with equal success; but in most of them, adds M. De Lacaille, "the cure was so rapid that, notwithstanding my long experience, I have asked myself if they could really have been yellow fever. Called in at the period of incubation, the triumph is easy."—Cameron, Social Science Congress.—*N. Y. Med. Times.*

**Pruritus Ani.**—For twenty years I have suffered from that annoying disease, pruritus ani, and had placed myself under the care of various physicians at different times, but without the least benefit. About two years ago, I noticed a brief paragraph in the Medical News, to the effect that balsam Peru would relieve that trouble. I tried it: and it gave immediate and entire relief. The disease still clings to me; but, when it becomes annoying, a single application of the balsam affords relief. Of all the medicines which I have tried, this is the only one that has had any effect.—*Cin. Lancet and Clinic.*

**Jamaica Dogwood.**—A writer remarks: I employed it with success in several cases in which opium could not be tolerated. The necessity for an anodyne in these cases arose, respectively, from the pain following an operation, from idiopathic inflammation, and from neuralgia. I should regard its anodyne properties, however, rather less powerful than those of opium, although its hypnotic action is more decided. I believe it will take rank between chloral and opium for its anodyne and hypnotic properties, while it is devoid of objections peculiar to each of these drugs. Dose, half to one drachm.—*Therapeutic Gazette.*

**Treatment of Gonorrhœa.**—Mr. W. Watson Cheyne, Asst. Surg. King's Coll. Hosp., in British Medical Journal, states that a bougie composed of fifty grains of iodoform, ten minims of oil of eucalyptus, and eighty-five grains of cocoa-butter passed into the urethra and secured by pad and strap at the meatus, allowed to dissolve and followed by an injection of boracic acid solution ("saturated solution of boracic acid") or an emulsion of eucalyptus oil "(one ounce of eucalyptus oil, one ounce of gum acacia, water to 40 or 20 ounces) to be used for two or three days," will effect a cure in ten days.—*Canada Med. Record, Oct. 1881.*

**Incompatibility of Salicylate of Sodium with Spirits of Nitrous Æther.**—A. W. Gerrard (Pharm. Journal and Trans., Nov. 8, 1881,) states that his attention has been called to a chemical reaction not generally known to prescribers, viz: that between salicylate of sodium and sweet spirits of nitre. The mixture gradually assumes a dark color, becoming at length nearly black, and letting fall a black deposit. The change takes place more rapidly in the light, and is brought about immediately by heating the mixture, although in that case the color does not become so dark. Together with the change in color there is developed an odor resembling that of gaultheria, so it is probable that there is formed in the reaction sodium nitrite and ethyl salicylate, but there must be also more complex changes to account for the darkening of the mixture.—*Detroit Lancet.*

**Beef Tea and Coca.**—At Charity Hospital, New York, Miss Corson said:

When a nutrient tonic is required, beef tea with coca infusion is one of the best known to the medical profession. Coca leaves, which come from South America, can be purchased at any large drug store. The coca is used to sustain strength and to stimulate the system when it is exhausted. To make the tonic, pour a half pint of boiling water on an ounce of dried coca leaves, and let it steep for an hour, keeping the water hot, but not boiling. Strain the coca infusion, and mix in equal parts with the beef tea, and the tonic is ready for the patient. It has a very unpleasant taste, judging from the faces of some of the ladies who ventured to introduce it into their mouths, but it is very effective.

**Giteau's** body is to have a thorough post mortem performed upon it, with a view to settling the question of insanity. We are glad they are to settle it in this way. If all murderers who plead the insanity dodge, were only hung, and then the question of responsibility settled by autopsy, there would be fewer murders. Do so some more, Messrs. Lawyers.—*Ex.*

**Cause of the Decay of Teeth.**—In a recent work by A. Weil, ("Zur Ætiologie der Infectious-krankheiten"), says Nature, the cause of the decay of teeth, whether external or internal, is stated to be the schizomycetous fungus, Leptothrix buccalis. The fungus can readily be detected by its acid reaction.—*N. Y. Med. Times.*

**Chloral Enemata in Vomiting of Pregnancy.**—Dr. Vidal (Paris Medicale) claims to have overcome vomiting of pregnancy by injecting 15 grains of chloral hydrate in infusion of orange leaves, twice daily. Dr. Dussand, of Marseilles, also claims good results from the same treatment.—*Chicago Med. Review.*

**Expert's Fees in Courts of Justice.**—The Supreme Court of Boston has decided that a physician is not bound to give his professional opinion for nothing, and that he can claim payment as an expert.—*Boston Med. and Surg. Reporter.*

